# Which PASI Outcome Is Most Relevant to the Patients in Real-World Care?

**DOI:** 10.3390/life11111151

**Published:** 2021-10-28

**Authors:** Natalia Kirsten, Stephan Rustenbach, Ralph von Kiedrowski, Christina Sorbe, Kristian Reich, Matthias Augustin

**Affiliations:** 1 Institute for Health Services Research in Dermatology and Nursing (IVDP), University Medical Center Hamburg-Eppendorf (UKE), Martinistraße 52, 20246 Hamburg, Germany; s.rustenbach@uke.de (S.R.); c.sorbe@uke.de (C.S.); k.reich@uke.de (K.R.); m.augustin@uke.de (M.A.); 2 Dermatology Office and Dermatology Study Center (CMSS) Dr. Ralph von Kiedrowski, Selters/Westerwald, Kirchstraße 1, 56242 Selters, Germany; dr.ralph.vonkiedrowski@praxis-kiedrowski.de

**Keywords:** psoriasis, outcomes measurement, preferences, benefits, registry

## Abstract

In psoriasis treatment, there is a high need to define meaningful endpoints and differences from the patient perspective to analyze patient-relevant differences of frequently used outcome methods for psoriasis under real-world conditions. A sample of 3116 patients from the German Psoriasis-Registry PsoBest was analyzed for clinical as well as patient-reported outcomes (PRO) after 3- and 6-month treatment. The parameters PASI, DLQI, and PBI were intercorrelated and related to two anchoring variables: (1) patient satisfaction with treatment and (2) perceived complete clearance. Baseline data were as follows: PASI 10.5 ± 9.1, DLQI 12.4 ± 3.4, and PBI 2.7 ± 0.3. There was an almost linear relationship between “complete patient satisfaction” and the relative differences in PASI in the range from PASI 25 to PASI 90. However, there was no additional benefit between PASI 90 and PASI 100. The same finding resulted from the anchoring variable “perception of complete healing”. When related to DLQI outcomes, relative PASI changes as well as absolute changes and PASI at 3 and 6 months showed relevant differences between the PASI classes 25 to 90 but not between PASI 90 and PASI 100. Under real-world conditions, changes in PASI and DLQI reflect patient-relevant benefits.

## 1. Background

Psoriasis is a chronic inflammatory skin disease manifested by the appearance of erythematous, scaly plaques mainly on the extensor sides. However, the whole body can be affected. Psoriasis can also occur on the joints, which is the case in about 30% of those affected [1,2]. Numerous other concomitant diseases, such as depression, cardiovascular, and metabolic diseases, are associated with psoriasis [3,4]. This leads to a severe reduction in the quality of life for sufferers [5,6]. 

Measuring treatment outcomes in psoriasis has become increasingly important for clinical research, clinical care, health services research, and health regulation [7]. Valid outcome tools are needed for the assessment of clinical efficacy [8], the use of treatment goals [9], and the evaluation of health technologies [10,11]. 

Most international guidelines recommend the combined use of both an objective measurement and a subjective assessment from the patient’s perspective [12]. Among patient-reported outcomes (PRO), health-related quality of life (HRQoL) is the most commonly used methodology [13] measured with standardized instruments. The validity and interpretability of such measurements are of critical importance [14,15]. 

To date, there is no general consensus on the preferred use of optimal outcomes parameters for psoriasis. This is due to (1) the lack of valid comparative data to existing instruments and (2) a general consensus on the required characteristics of an ideal instrument. 

Although the Psoriasis Area and Severity Index (PASI) [16,17] and the Dermatology Life Quality Index (DLQI) [18,19] have not been validated and further developed in the manner required today, they have emerged as the by far the most widely used outcome instrument for objective measurement and patient-reported outcomes in psoriasis, respectively. The large number of clinical trials as well as non-interventional studies may have been the reason that regular authorities, such as the Food and Drug Administration (FDA) [20] and the European Medicines Agency (EMA) [21], primarily accept these instruments as outcome measures that reflect efficacy, effectiveness, and patient benefit. 

While PASI and DLQI are of fundamental value, they do not reflect patient preferences and therefore cannot be used as a tool for treatment selection. To broadly integrate patient preferences and needs, the Patient Benefit Index (PBI) was developed and validated. In contrast to DLQI, PBI is an individualized and goal-oriented instrument, but it can hardly be used in simple clinical care. 

The development of new targeted therapies for psoriasis, such as IL-17 inhibitors or IL-23 inhibitors, showed a clear superiority in efficacy compared to older therapies. This led to a rethinking of the primary endpoints so that in most clinical trials on the efficacy of new targeted therapies, PASI 90 response was given as primary endpoint [22,23,24]. Since there is a large number of limitations of PASI and DLQI and since some of their characteristics are more relevant to routine care than to the highly selected patient populations in clinical trials, the present study was conducted to assess the clinical significance of positive outcomes for psoriasis under routine conditions in relation to DLQI, PBI, and single-anchor variables.

The key questions were as follows:

1. Which relative PASI outcomes and which DLQI endpoints are associated with maximum patient-reported outcomes in psoriasis routine care?
2. To what extent are PASI 75, PASI 90, and PASI 100 differentiated after three and six months of treatment?

## 2. Methods

### 2.1. Patients

Patient data included in the study were obtained from the German Psoriasis-Registry (PsoBest). All patients enrolled from the start of the registry in January 2008 until the approval of IL-17 inhibitors were included if the following inclusion criteria were met: age over 18 years, presence of moderate or severe plaque-type psoriasis, initial initiation of systemic therapy (conventional and biologics), and the ability to provide written informed consent to participate in the study. 

### 2.2. Outcomes

Visits were conducted at 3-month intervals for the first 6 months, and patient-reported outcomes were recorded in addition to the clinical parameters (Table 1).

### 2.3. Statistical Analysis

The association between clinical response (measured by the physician), patient benefit (measured by the Patient Benefit Index), and the predefined anchor variables “all lesions healed” and “satisfaction” were assessed. For the variable “satisfaction,” there were three response options: very dissatisfied, rather not satisfied, and very satisfied. 

The anchor variable “all lesions healed” is an item from the PBI. To determine patient benefit, the PBI consists of two parts. The first part (T1) is used at the beginning of treatment and the second part during the course of therapy (T2). At T1, the importance of patient needs is assessed, and at T2, the achievement of these needs is determined by a second survey. The patient can indicate values between 0, “goal not achieved at all,” and 4, “goal achieved to the maximum”. The anchor variable “all lesions healed” corresponds to the fourth item of the second part of the questionnaire, which was rated 4 (PBQ4) by patients who maximally achieved this specific goal.

Descriptive cross-tabulation was performed to represent associations.

Predictors of PASI response and achievement on the anchor variables were tested by logistic regression controlling for age and gender. Multiple regression analysis was performed for the DLQI.

Only data sets containing complete documentation of the variables of interest were included in the analysis.

All analyses were conducted using IBM SPSS Statistics for Microsoft Windows version 18.

## 3. Results

### 3.1. Patients

A total of 3116 patients with moderate to severe psoriasis were analyzed, including 58% men, mean age 48 ± 31 years, mean disease duration 18 years, 31% having psoriatic arthritis, and 56% with naive psoriasis. A total of 74% were started on systemic treatment, 26% with biologics. The mean PASI at baseline was 10.5 ± 9.1; the mean DLQI was 12.4 ± 3.4. At three months, mean outcomes were PASI 5.2 (±7.3) and DLQI 2.5 (±2.8) and at six months, PASI 4.9 (±6.3) and DLQI 2.4 (±2.2).

### 3.2. Cross-validation by Anchoring Variables

**Figure 1:**
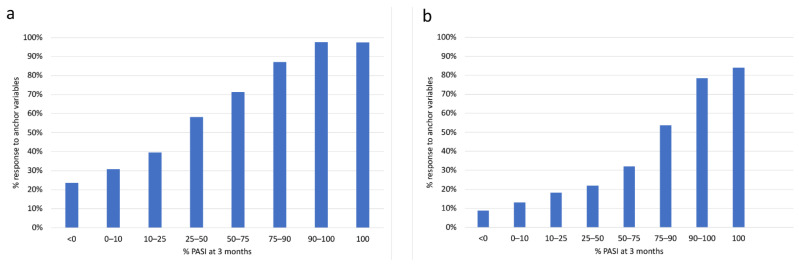
Proportion of patients satisfied with treatment outcome (**a**) and perceiving “all lesions healed,” (**b**) grouped by Psoriasis Area and Severity Index (PASI) response at 3 months (n = 2534 patients).

There was a clear association between increasing PASI response groups and the anchor variable “patient completely satisfied” (Figure 1a). Especially in the range of PASI 50, PASI 75, and PASI 90, the proportion of patients who were satisfied with the treatment increased largely linearly. In contrast, there was no clear difference at a high level between PASI 90 and PASI 100. There was also an almost linear increase between PASI 50 and PASI 90 in patients’ perception of being healed of all skin lesions (Figure 1b). Again, there were no relevant differences between PASI 90 and PASI 100. Remarkably, even in the group with PASI 100, only about 80% of patients felt that their skin lesions had been completely removed. 

### 3.3. Characterization of PASI by Patient-Reported Outcomes

**Figure 2:**
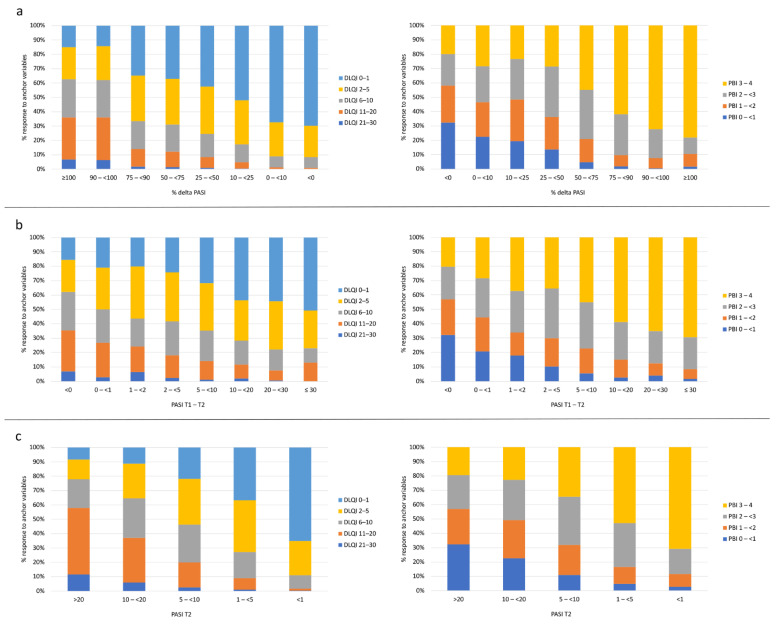
Association of Psoriasis Area and Severity Index (PASI) outcomes % delta PASI (**a**), absolute PASI difference T1–T2 (**b**), and the resulting PASI at T2 (**c**) related to DLQI classes at T2 (left column) and to Patient Benefit Index (PBI) groups at T2 (right column). T1 refers to baseline and T2 to 3 months follow-up.

When analyzing PASI outcomes by different DLQI classes, there was a shift with increasing PASI response toward more patients in DLQI classes 0–1 and 2–5 (Figure 2). Using relative improvements in PASI, there was no difference between PASI 90 and PASI 100 and the distribution of DLQI. The distribution profiles were relatively similar when using relative PASI improvement, absolute PASI difference, or absolute PASI at three months as outcomes. A similar differentiation was observed when PBI rather than DLQI classes were used for analysis. All relationships between PASI, DLQI, PBI, and the anchoring variables did not differ significantly between the three-months and six-months measurement time points, as shown by the curves between PASI and PBI.

### 3.4. Association of the Relationship between PASI/DLQI and Anchor Variables to Baseline

Regression analyses showed that the PASI at baseline significantly influenced all outcome parameters at T2 and T3 except for the anchor variable “satisfaction” at T3 (six months). Controlling for age and gender, each one-point increase in PASI at baseline increased the chance of achieving PASI 75 and PASI 90 by approximately 4%.

Controlling for PASI at baseline and age, female patients had about a 26% higher chance of achieving PASI 75 at month 6 and about 40% higher chance of achieving PASI 90 at month 3 and about 54% at month 6.

Controlling for PASI at baseline and gender, increasing age was a predictor of achieving PASI 75 and PASI 90 at month 6. In addition to clinical outcomes measured by PASI, increasing age was associated with a higher satisfaction at month 3, being healed of all lesions at months 3 and 6, and lower impairment in health-related quality of life (HRQoL) at months 3 and 6. On average, the probability of these effects increased by approximately 1% with each year of age (Table 2).

## 4. Discussion

In order to characterize differences in PASI outcomes in this study, two anchor variables were applied: one reflecting the extent of patient satisfaction with the treatment outcome and the second reflecting the patients’ perception of completely healed skin lesions. However, in this real-world population of patients and physicians, no obvious differences were found between PASI 90 and PASI 100 from the patients’ perspective. This finding is supported by the association of PASI and DLQI. Again, differences were observed in PASI 50, 75, and 90, whereas losses in PASI 90 and PASI 100 made no difference in the DLQI distribution. The finding of an association between relative delta PASI and both anchor variables supports the use of relative PASI improvements as patient-relevant outcomes in general. 

Our data are consistent with data from the literature showing that there is little concordance between DLQI and PASI in a cross-sectional setting, reflecting a discrepancy between objective and subjective perception of skin disease [25,26,27]. In contrast, changes after treatment correlate markedly [28,29].

Another important finding is the fact that a significant proportion of patients reaching PASI 100 shows no signs of satisfaction and of receiving completely cleared skin in the anchoring variables. This observation requires further explanation since it provides a new perspective on a discrepancy between physician and patient understanding of complete clearance. One explanation could be that physicians in real-world conditions, even if experienced in PASI evaluation, have lower accuracy in PASI measurement than study physicians in controlled clinical trials. Another explanation could be the lack of validity of the PASI at low baseline values. Last but not least, it is possible that certain comorbidities, such as psoriasis arthritis and its associated symptoms, may cause discomfort to the patient and affect his or her perception of not feeling completely symptom-free.

The regression analysis could show that the baseline PASI was a predictor of clinical response (PASI 75/PASI 90) as well as patient-reported outcomes. Age and gender were thus important factors influencing the achievement of higher PASI response rates and satisfaction. 

Taken together, these results indicate that

(1) percentage PASI response and absolute target DLQI are visible but not optimum outcomes for measuring treatment success in psoriasis systemic treatment.
(2) PASI 90 and PASI 100 reflect larger patient benefits from treatment than lower numbers.
(3) PASI 90 and PASI 100 do not differentiate under real-world conditions of psoriasis care in Germany.
(4) any improved PASI class between PASI 25 and PASI 90 as well as between DLQI > 20 and DLQI < 2 provides added value to the patients.
(5) in patients reaching less than PASI 75, still a marked group reaches good quality of life (DLQI 0–1) and high level of satisfaction with treatment.

Since an antipsoriatic drug with a high mean PASI 75 or PASI 90 response from clinical trials is more likely to induce a relevant gain in quality of life and patient benefit, the PASI response may be a valid surrogate for treatment decisions under real-world conditions. Nevertheless, due to the limitations outlined previously, single PASI responses in practice do not provide sufficient information about patient-relevant therapeutic benefits in the real-world setting. For this reason, a patient-reported outcome must be included in the definition and follow-up of therapeutic goals.

Limitations of the current study lie in the real-world environment, where the accuracy of outcome measurement is less controllable than in clinical trials. Nevertheless, the study was intentionally conducted in the PsoBest registry. Patients are still in a controlled data evaluation with a quality assurance program, but they are intentionally treated under real-world conditions. Another limitation is the heterogeneity of patients at baseline, again reflecting routine care rather than clinical trial settings. Low baseline PASI scores may therefore comprise the internal validity of PASI measurement while increasing external validity.

## Figures and Tables

**Table 1:** Flowchart of treatment outcomes and time points in the data set from the German Psoriasis-Registry PsoBest (n = 3116).

Parameter	T1	T2	T3
	Inclusion	3 Months	6 Months
		Primary Time Point	Secondary Time Point
General			
Patient socioeconomic data	X		
Clinical history	X		
Clinical Outcomes			
PASI	X	X	X
PGA	X	X	X
BSA	X	X	X
Patient-Reported Outcomes			
DLQI	X	X	X
PBI	X	X	X
Anchoring Variable: Satisfaction		X	X
Anchoring Variable: Complete Healing		X	X

DLQI, Dermatology Life Quality Index; PBI, Patient Benefit Index; PGA, Physician Global Assessment; BSA, body surface area; PASI, Psoriasis Area and Severity Index.

**Table 2:** Regression analysis at months 3 and 6. Association of the relationship between Psoriasis Area and Severity Index (PASI)/Dermatology Life Quality Index (DLQI) and anchor variables at baseline.

**PASI 75 Achieved after 3 Months**					
**Omnibus Test**	**n**	**PASI 75 Achieved**	**Chi-square**	**df**	**Sig.≤**
	2240	756	82.008	3	**0.000**
**Baseline Predictors**	**B**	**SE**	**Exp(B)**	**df**	**Sig.≤**
Sex	0.169	0.093	1.184	1	0.069
Age	0.006	0.003	1.006	1	0.087
PASI	0.041	0.005	1.042	1	**0.000**
Constant	−1.629	0.183	0.196	1	**0.000**
**PASI 90 Achieved after 3 Months**					
**Omnibus Test**	**n**	**PASI 90 Achieved**	**Chi-square**	**df**	**Sig.≤**
	2240	325	53.662	3	**0.000**
**Baseline predictors**	**B**	**SE**	**Exp(B)**	**df**	**Sig.≤**
Sex	0.339	0.123	1.404	1	**0.006**
Age	0.007	0.004	1.007	1	0.118
PASI	0.039	0.006	1.040	1	**0.000**
Constant	−2.867	0.246	0.057	1	**0.000**
**“Satisfaction” Achieved after 3 Months**					
**Omnibus Test**	**n**	**“Satisfaction” Achieved**	**Chi-square**	**df**	**Sig.≤**
	2229	1096	19.830	3	**0.000**
**Baseline Predictors**	**B**	**SE**	**Exp(B)**	**df**	**Sig.≤**
Sex	−0.028	0.087	0.973	1	0.751
Age	0.008	0.003	1.008	1	**0.010**
PASI	0.016	0.004	1.016	1	**0.000**
Constant	−0.629	0.170	0.533	1	**0.000**
**“All Leasons Healed” Achieved after 3 Months**					
**Omnibus Test**	**n**	**PNQ 4 Achieved**	**Chi-square**	**df**	**Sig.≤**
	2120	391	14.572	3	**0.002**
**Baseline Predictors**	**B**	**SE**	**Exp(B)**	**df**	**Sig.≤**
Sex	0.100	0.114	1.106	1	0.381
Age	0.014	0.004	1.014	1	**0.001**
PASI	0.008	0.006	1.008	1	0.167
Constant	−2.325	0.231	0.098	1	**0.000**
**DLQI after 3 Months**					
**Omnibus Test**	**n**	**df**	**F**	**Sig.≤**	
	2240	3	15.458	**0.000**	
**Baseline Predictors**	**B**	**SE**	**Beta**	**t**	**Sig.≤**
Constant	6.138	0.472		13.013	**0.000**
Sex	0.972	0.244	0.084	3.979	**0.000**
Age	−0.037	0.009	−0.091	−4.342	**0.000**
PASI	0.049	0.012	0.084	3.981	**0.000**
**PASI 75 Achieved after 6 Months**					
**Omnibus Test**	**n**	**PASI 75 Achieved**	**Chi-square**	**df**	**Sig.≤**
	1933	919	100.645	3	**0.000**
**Baseline Predictors**	**B**	**SE**	**Exp(B)**	**df**	**Sig.≤**
Sex	0.231	0.096	1.260	1	**0.016**
Age	0.012	0.003	1.012	1	**0.000**
PASI	0.045	0.005	1.047	1	**0.000**
Constant	−1.453	0.193	0.234	1	**0.000**
**PASI 90% Achieved after 6 Months**					
**Omnibus Test**	**n**	**PASI 90 Achieved**	**Chi-square**	**df**	**Sig.≤**
	1933	500	78.066	3	**0.000**
**Baseline Predictors**	**B**	**SE**	**Exp(B)**	**df**	**Sig.≤**
Sex	0.433	0.108	1.543	1	**0.000**
Age	0.011	0.004	1.011	1	**0.005**
PASI	0.040	0.005	1.040	1	**0.000**
Constant	−2.378	0.221	0.093	1	**0.000**
**“Satisfation” Achieved after 6 Months**					
**Omnibus Test**	**n**	**“Satisfaction” Achieved**	**Chi-square**	**df**	**Sig.≤**
	1905	1030	3.043	3	0.385
**Baseline Predictors**	**B**	**SE**	**Exp(B)**	**df**	**Sig.≤**
Sex	0.041	0.094	1.042	1	0.663
Age	0.003	0.003	1.003	1	0.305
Pasi	0.006	0.005	1.006	1	0.167
Constant	−0.113	0.185	0.893	1	0.541
**“All Leasons Healed” Achieved after 6 Months**					
**Omnibus Test**	**n**	**PNQ 4 Achieved**	**Chi-square**	**df**	**Sig.≤**
	1831	389	15.530	3	**0.001**
**Baseline Predictors**	**B**	**SE**	**Exp(B)**	**df**	**Sig.≤**
Sex	0.163	0.117	1.177	1	**0.163**
Age	0.010	0.004	1.010	1	**0.013**
PASI	0.016	0.005	1.016	1	**0.004**
Constant	−2.115	0.235	0.121	1	**0.000**
**DLQI after 6 months**					
**Omnibus test**	**n**	**df**	**F**	**Sig.≤**	
	1920	3	6,173	**0.000**	
**Baseline predictors**	**B**	**SE**	**Beta**	**t**	**Sig.≤**
Constant	5.219	0.487		10.723	**0.000**
Sex	0.257	0.249	0.024	1.030	0.303
Age	−0.029	0.009	−0.074	−3.258	**0.001**
PASI	0.033	0.012	0.061	2.681	**0.007**

B, regression coefficient unstandardized; Beta, regression coefficient standardized; SE, standard error of the coefficient; Exp(B), odds ratio; df, degrees of freedom; F, F-Value; Sig., Level of significance; t, t-value; PNQ 4, 4th item of the second part of the PBI.

## Data Availability

Data is publicly not available. There are restrictive registry rules for access. Moreover, European legislation as well as informed consent given prohibits the sharing of medical and health data from the registry for privacy issues. An anonymization of detailed medical and health records is not possible.
